# The mediating effect of alexisomia between childhood abuse and depression/stress in a sample of Lebanese adults: A cross-sectional study

**DOI:** 10.1017/gmh.2026.10275

**Published:** 2026-07-29

**Authors:** Christian Salameh, Diana Malaeb, Muna Barakat, Fouad Sakr, Mariam Dabbous, Souheil Hallit, Feten Fekih-Romdhane, Sahar Obeid

**Affiliations:** 1 Holy Spirit University of Kaslik, Lebanon; 2 Gulf Medical University, United Arab Emirates; 3 Applied Science Private University, Jordan; 4 Lebanese International University, Lebanon; 5 Holy Spirit University of Kaslik Faculty of Medicine and Medical Sciences, Lebanon; 6 Université de Tunis El Manar, Tunisia; 7 Lebanese American University-Byblos Campus, Lebanon; 8 Razi Hospital, Manouba, Tunisia

**Keywords:** alexisomia, depression, stress, childhood abuse, Lebanon

## Abstract

Mental health outcomes, such as depression and stress, are shaped by various factors, including childhood abuse, emotional and physical awareness deficits like alexisomia and sociodemographic influences. This study aimed to investigate the mediating role of alexisomia in the relationship between childhood abuse and mental health outcomes, specifically depression and stress, among Lebanese adults. A cross-sectional study was conducted in July 2024, enrolling 1,205 participants via a snowball sampling technique from various Lebanese regions. Higher childhood abuse was significantly associated with greater alexisomia, depression and stress. Mediation analyses with adjustment for covariates (gender, smoking, socioeconomic status, etc.) revealed that alexisomia mediated the association between childhood abuse and both depression (Beta = 0.018; Boot CI: 0.002–0.034) and stress (Beta = 0.005; Boot CI: 0.001–0.009). Higher childhood abuse was significantly associated with higher alexisomia and directly associated with higher depression/stress. Higher alexisomia was significantly associated with higher depression/stress. The findings suggest the need for greater clinical focus and targeted interventions to enhance emotional and physical awareness and improve mental health outcomes, particularly for individuals with a history of trauma.

## Impact statement

This study highlights an important pathway through which childhood abuse affects mental health in adulthood by identifying alexisomia – difficulties in recognizing and interpreting bodily and emotional signals – as a key mediating factor. By clarifying this mechanism, the findings go beyond documenting associations and help explain how early trauma translates into higher levels of depression and stress later in life. The results have meaningful implications for clinical practice. They suggest that mental health problems linked to childhood abuse are not only emotional in nature but also involve disrupted mind–body awareness. Interventions that strengthen emotional and physical awareness, such as mindfulness-based or body-focused approaches, may therefore complement traditional psychological treatments and improve outcomes for individuals with trauma histories. At a broader level, this study provides locally relevant evidence from a Lebanese adult population, a context marked by multiple stressors and limited mental health resources. The findings support the adoption of trauma-informed and culturally sensitive care models and underscore the value of preventive and early interventions targeting awareness and coping. Overall, this research contributes to a more integrative understanding of mental health by linking early adversity, bodily awareness and psychological well-being.

## Introduction

With their growing prevalence (Hoying et al., 2020; Dagher et al., 2023), depression and stress are two major public health challenges associated with substantial functional impairment (Tenk et al., 2018; Gutiérrez-Rojas et al., 2020; Maier et al., 2021; WHO, 2023a). Depression, as defined by the WHO, is a persistent low mood, anhedonia and/or diminished interest in activities, leading to significant impairments in daily functioning (WHO, 2023a). Stress, on the other hand, is a multifaceted phenomenon often described as a state of mental tension or worry caused by adverse circumstances (WHO, 2023b). Conceptually, stress arises when environmental demands exceed an individual’s capacity to cope, posing a threat to well-being (Mathur et al., 2016). Both conditions share overlapping risk factors and mechanisms (Hoying et al., 2020). Because of these shared mechanisms, studying depression and stress together is relevant for their interconnected nature. A key shared risk factor for both depression and stress is exposure to violence or trauma, particularly during the critical developmental period (Ranasinghe et al., 2017; Hoying et al., 2020). Childhood abuse has repeatedly been associated with heightened depression and stress levels in adults (Gutiérrez-Rojas et al., 2020; Humphreys et al., 2020; Thurston et al., 2023).

### Childhood abuse, depression and stress

Childhood abuse, defined as any harmful mistreatment of individuals under 18 years of age that causes physical or psychological harm (WHO, 2024), is a well-documented risk factor for both depression and stress. Globally, an estimated one billion children endure abuse annually (Hillis et al., 2016; El Zouki et al., 2024), which falls into four main categories: physical, sexual, emotional abuse and neglect (WHO, 2024). All of these forms are associated with a series of adverse effects in adulthood, including severe psychological distress (Thurston et al., 2023). They disrupt emotional and cognitive development, increasing vulnerability to mood disorders and chronic stress responses in adulthood (Lang et al., 2020). The link between childhood abuse and depression and stress is well-established in the literature (Li et al., 2020). Systematic reviews (Li et al., 2020), meta-analyses (Humphreys et al., 2020; Mandelli et al., 2015; Watters et al., 2021) and both retrospective (Berber Çelik and Odacı, 2020) and longitudinal studies (Thurston et al., 2023) demonstrated a strong association between childhood abuse and the risk of depression over the lifespan. A meta-analysis including 184 studies and a total of 255 effect sizes demonstrated that individuals who experienced childhood abuse are 2.66–3.73 times more prone to developing depression, with emotional abuse showing the strongest predictive value for depression severity compared to physical or sexual abuse (Nelson et al., 2017). In survivors, this often manifests as earlier onset of depression, recurrent episodes and resistance to treatment (Humphreys et al., 2020).

Similarly, childhood abuse is strongly associated with heightened stress. A systematic review linked childhood abuse to disruption of the hypothalamic–pituitary–adrenal (HPA) axis, leading to prolonged stress and increased vulnerability to stress-related disorders, including post-traumatic stress disorder (Lang et al., 2020). Childhood abuse was also found to negatively impact self-esteem, which in turn heightened stress levels (Berber Çelik and Odacı, 2020). Childhood abuse has also been associated with complex post-traumatic stress disorder (CPTSD), a condition characterized by disturbances in self-organization, affect regulation difficulties and negative self-concept, which frequently co-occurs with depression and other trauma-related symptoms. In that sense, traumatic stress can be viewed as not only an outcome of abuse but also as a pathway contributing to depression, in part through HPA disruption (Spinhoven et al., 2014; Murphy et al., 2022).

### Childhood abuse, alexisomia, depression and stress

Various social and psychological mediators have been studied to understand how childhood abuse leads to depression and/or stress. This study focuses on alexisomia as a potential mediator in these relationships. Emotional dysregulation, one of the most extensively studied mediators, has been shown to disrupt a person’s ability to process and regulate emotions effectively, creating a pathway to both depression and stress (Christ et al., 2019). Within this context, alexithymia, a typical trait of lacking emotional awareness, has received special attention linking childhood abuse and psychopathologies (Kanbara and Fukunaga, 2016). Against this background, alexisomia – characterized by a difficulty in identifying and describing somatic sensations and experiences such as hunger, fatigue, pain, and so forth (Kanbara and Fukunaga, 2016; Oka, 2020) – emerges as a compelling, relatively new factor. The concept originates from psychosomatic research and highlights impaired bodily awareness (Oka, 2020). While alexithymia concerns deficits in emotional awareness, alexisomia concerns deficits in somatic awareness, making it possible to conceptualize alexisomia as the somatic counterpart of alexithymia. As shown by evidence from the literature, trauma-related psychopathology extends beyond emotional awareness, and childhood abuse affects both emotional and somatic awareness (Kanbara and Fukunaga, 2016; Oka, 2020). There is an association between early experiences of trauma and somatic problems, alexithymia and distress (Güleç et al., 2013). Additionally, earlier studies have revealed that processes related to alexithymia can partly mediate the relationship between childhood abuse and subsequent symptoms (Kanbara and Fukunaga, 2016).

Alexisomia encompasses several interconnected concepts: Overadaptation (OA), Difficulty Identifying Bodily feelings (DIB) and Lack of Health Management based on bodily feelings (LHM). OA captures the tendency to prioritize external social expectations over personal bodily experiences, often suppressing internal cues to conform to societal norms (Oka, 2020). DIB reflects an inability to recognize bodily signals related to stress regulation and homeostasis, such as fatigue, hunger or tension, whether these signals are normal or disordered (Ikemi and Ishikawa, 1979; Kotov et al., 2017). LHM highlights the failure to appropriately respond or act on bodily sensations in daily health practices, which can lead to inadequate medical attention or self-care (Ikemi and Ishikawa, 1979; Oka, 2020). This condition is particularly relevant in trauma survivors, where it has been shown to exacerbate mental health symptoms (Oka, 2020). Research suggests that survivors of childhood abuse experience significantly higher somatic complaints and difficulties in emotional processing, which are closely tied to alexisomia (Luoni et al., 2018; Khan and Jaffee, 2022). Alexisomia is also known to be notably prevalent among individuals with stress-related chronic pain, reinforcing its role in the stress response pathway (Luoni et al., 2018; Xu et al., 2024). The inability to identify and articulate sensations – key components of alexisomia – has been found to exacerbate emotional dysregulation, hinder coping mechanisms and amplify the physiological stress response (Xu et al., 2024). These challenges reflect disruptions in the body’s ability to manage emotional and stress-related signals, contributing to both depression and stress (Kotov et al., 2017; Khan and Jaffee, 2022), thus potentially creating a pathway from abuse to depression and stress (Xu et al., 2024). However, some studies failed to show a significant link between alexithymia and psychiatric disorders, including major depressive disorder (Honkalampi et al., 2007).

Although several mediators have been studied to explain the above-mentioned relationship, including coping strategies, personality types and social support (Fritz et al., 2018; Christ et al., 2019), growing evidence suggests that trauma-related psychopathology also involves disturbances in interoception and bodily self-awareness (Khan and Jaffee, 2022). Within this framework, studying alexisomia as a mediator could enhance our understanding of the mechanisms underlying psychosomatic and emotional dysfunction in childhood trauma survivors (Khan and Jaffee, 2022). While alexithymia has been studied in trauma patients’ psychopathology, the contribution of the impaired bodily awareness, commonly found in this population, remains understudied (Schmitz et al., 2023). Emerging evidence supports the role of interventions targeting interoceptive awareness in improving psychological functioning in trauma-related conditions (Heim et al., 2023). Therefore, examining the role of bodily awareness in the mechanism of psychopathology becomes clinically relevant. Alexisomia may also represent a potentially modifiable target for intervention (Oka and Lkhagvasuren, 2022; Xu et al., 2024). Therapeutic approaches that improve emotional awareness and somatic processing could reduce some of the adverse consequences of childhood abuse (Oka and Lkhagvasuren, 2022; Xu et al., 2024). These strategies could complement existing therapies focused on emotional regulation, creating a more comprehensive framework to address the psychological aftermath of trauma (Li et al., 2020).

### The present study

In Lebanon, sociopolitical instability, compounded by economic crises, has amplified childhood abuse rates and mental health challenges. UNICEF reports that 57% of children in Lebanon endure severe violence, and over 90% of households with children struggle to meet basic needs (United Nations Children’s Fund (UNICEF), 2024). This precarious environment not only exacerbates childhood abuse but also limits access to adequate mental health care, highlighting the urgency of tackling this issue. On the other hand, Lebanese studies reveal alarming rates of mental health issues. For instance, studies show that the prevalence of alexithymic behaviors, aggression and depression among Lebanese young adults is notably high compared to global figures (31.7% exhibited alexithymia, and 26% had severe depression) (Abdo, 2016; Sfeir et al., 2020). Other research found a significant correlation between exposure to violence and heightened stress levels among young Lebanese adults (Itani et al., 2018). Additionally, the compounded effects of child physical injury and chronic socioeconomic stressors have been shown to significantly increase the prevalence of post-traumatic stress disorder (PTSD) and depression in the Lebanese population (Maalouf et al., 2022).

Moreover, the cultural stigma surrounding emotional and psychological discussions in Lebanon may intensify alexisomia, restricting survivors’ ability to articulate their distress and seek help effectively (Mathews et al., 2020). These findings underscore the need for a better understanding of the relationship between these conditions, as well as for targeted interventions to address both the systemic and cultural barriers contributing to childhood abuse and mental health challenges in Lebanon. Therefore, the objective of the study is to tackle the mediating role of alexisomia in the relationship between childhood abuse and mental health outcomes, specifically depression and stress, among a sample of Lebanese adults.

## Methods

### Study design

The cross-sectional study was conducted in July 2024, with a total of 1,205 participants enrolled using snowball sampling from multiple Lebanese governorates. Individuals were invited to participate via a questionnaire created on Google Forms. The study recruited participants using diverse platforms, such as social media networks, medical forums and academic communities. Participation was voluntary, and no incentives were provided. All participants were briefed about the study’s aims and guaranteed confidentiality before providing informed consent. The questionnaire contained a comprehensive description of the study’s purpose, confidentiality assurances and the consent form. Eligibility criteria included Arabic-speaking adults aged 18 years or older, capable of completing the survey in Arabic and willing to provide informed consent. Individuals with active substance abuse issues or severe psychiatric conditions (other than depression or stress) were excluded to minimize potential confounding effects on the measurement of alexisomia, depression and stress, as these conditions are known to be associated with elevated levels of alexithymia (Oka, 2020), depression and stress (Dagher et al., 2023).

The questionnaire underwent a pilot test with 30 Arabic-speaking participants to assess its clarity, cultural appropriateness and technical functionality. Based on feedback from the pilot group, minor revisions were made to improve the phrasing and enhance participants’ understanding of the questions. The pilot test data were excluded from the final dataset.

### Sample size calculation

According to the Fritz and MacKinnon formula (Fritz and Mackinnon, 2007), *N*= $\frac{L}{f^{2}}+k+1$ , a minimum sample size of 414 participants was required. This estimation is based on *L* = 7.85 corresponding to a 5% alpha error and 80% statistical power, *f* = 0.14 for a small effect size, and *k* = 12 predictors included in the model.

### Questionnaire and measures

Data were collected through an online, anonymous, self-administered questionnaire, which was made available in Arabic. The questionnaire was organized into two sections:

The first section collected basic sociodemographic data, including age, gender and educational background. The Household Crowding Index (HCI) (Melki et al., 2004) was also included as a measure of the family’s socioeconomic status. The HCI was determined by dividing the total number of household members by the total count of rooms (excluding bathrooms and the kitchen). This index serves as a valuable indicator of living conditions and was considered a potential confounder in the study’s analyses (Hoying et al., 2020). We also added questions related to the current smoking status and alcohol consumption.

The second section included scales:

**Childhood Abuse Severity Retrospective Scale (CASRS-12)** (Bernstein et al., 1994)**:** This 12-item scale assesses 4 dimensions of childhood abuse: sexual, physical, psychological abuse and neglect. It was validated in Arabic in the Lebanese population (Fekih-Romdhane et al., 2022) (*ω* = 0.87–0.93). Responses are reported on a 4-point Likert scale where 1 corresponds to “Never,” and 4 corresponds to “Frequently,” with higher scores meaning greater abuse severity. The questionnaire includes statements like “*I am beaten up because of every small mistake*” (Bernstein et al., 1994) (in this study, Cronbach’s *α* = 0.85).

**Shitsu-Taikan-Sho** **Scale (STSS)** (Arimura Tatsuyuki et al., 2012)**:** A self-report tool, validated in Arabic in Lebanon(Samaha et al., 2024), composed of 23 items and used to assess alexisomia (*ω* = 0.89/ *α* = .89). Developed in Japan, the scale has shown strong validity and reliability in both general and clinical populations. It includes three subscales: Overadaptation (OA), Difficulty Identifying Bodily feelings (DIB) and Lack of Health Management based on bodily feelings (LHM). The DIB subscale consists of nine questions, the OA subscale includes six questions and the LHM subscale contains seven questions. Participants respond to each item on a 5-point Likert scale where 1 corresponds to “Definitely false” and 5 to “Definitely true.” Sample statements from the questionnaire include “*I find it hard to identify how my body feels in certain situations*” (Arimura Tatsuyuki et al., 2012) (in this study, Cronbach’s *α* = 0.89).

**Patient Health Questionnaire-9 (PHQ-9)** (Kroenke et al., 2001)**:** This 9-item scale, validated in Arabic for the Lebanese population (Cronbach’s *α* = 0.88) (Sawaya et al., 2016; Dagher et al., 2023), is designed to assess the nine diagnostic criteria for major depressive disorder from the Diagnostic and Statistical Manual of Mental Disorders (DSM-IV) over the past 14 days. One of the items, “*thoughts that you would be better off dead or of hurting yourself in some way*,” is considered positive if present, regardless of duration. Each item is scored on a scale from 0 (no symptoms) to 3 (symptoms present nearly every day), resulting in a total score ranging from 0 to 27. Depression severity is categorized based on the total score, ranging from no depression to mild, moderate, moderately severe or severe depression. The scale evaluates an individual’s level of interest, mood, sleep quality, energy levels, eating habits, self-perception, concentration and the presence of suicidal thoughts (Kroenke et al., 2001) (in this study, Cronbach’s *α* = 0.89).

**Stress Numerical Rating Scale (SNRS-11)** (Karvounides et al., 2016)**:** This scale, validated in Arabic in Lebanon (Obeid et al., 2024), measures both momentary (state) and day-to-day perceived stress on a scale from 0 (“No stress”) to 10 (“Highest stress possible”). For momentary stress, participants rate their current stress level, while day-to-day stress reflects the past week. Participants answered the question, “*On a scale of 0 to 10, what number best describes your level of stress right now?.*” Scores are categorized as no stress (0), mild (1–3), moderate (4–7) or severe (8–10), focusing on stress intensity as one dimension of the broader stress construct. (*α* = 0.79–.87).

### Ethical considerations

This study followed the ethical principles outlined in the Declaration of Helsinki (World Medical Association, 2013). Before enrollment, all individuals submitted digital informed consent. The confidentiality and anonymity of participants’ data were strictly maintained throughout the research process.

### Statistical analysis

The SPSS v.27 software was used for the statistical analysis. Depression and stress scores were considered normally distributed since the skewness and kurtosis values were between the −1 and the +1 interval. Student’s *t*-test was used to compare two means, ANOVA test to compare three or more means and Pearson’s test to correlate two continuous variables. The mediation analysis was performed using PROCESS MACRO (an SPSS add-on) v3.4 Model 4, with the number of bootstrap samples set at 5,000 and a 95% confidence interval. Four routes result from this analysis: pathway A of the independent variable to the mediator, pathway B of the mediator to the dependent variable and pathways C and C′ indicating the total and direct effects of the independent variable to the dependent variable. We considered the mediation analysis to be significant if the confidence interval did not pass through zero. Covariates entered in the model were those that showed a *p* < 0.25 in the bivariate analysis. *p* < 0.05 was considered statistically significant.

## Results

In total, 1,205 participants were included in this study, with a mean age of 27.16 years (SD: 10.23) and 63.7% female. Other descriptive statistics of the sample can be found in Table 1.

**Table 1.** Sociodemographic and other characteristics of the sample (*N* = 1,205) Table 1. long description.

Variable	*N* (%)
Gender	
Male	437 (36.3%)
Female	768 (63.7%)
Education	
School level	188 (15.6%)
University level	1,017 (84.4%)
Current smoking	
No	766 (63.6%)
Yes	439 (36.4%)
Current alcohol drinking	
No	1,086 (90.1%)
Yes	119 (9.9%)
	**Mean ± SD**
Age (years)	27.16 ± 10.23
Household overcrowding index (person/room)	1.12 ± 0.59
Childhood abuse	10.17 ± 7.01
Depression	9.40 ± 6.17
Stress	4.73 ± 2.75
Alexisomia	49.26 ± 11.39

### Bivariate analysis of factors associated with depression and stress

Participants who smoked had significantly higher depression and stress scores. Females had significantly higher stress scores (Table 2). Furthermore, higher alexisomia and childhood abuse were significantly associated with higher depression and stress scores (Table 3). Higher childhood abuse was relatively weakly associated with higher alexisomia.

**Table 2.** Bivariate analyses of factors associated with depression and stress scores Table 2. long description.

	Depression score			Stress score		
	Mean ± SD	*p*	Effect size	Mean ± SD	*p*	Effect size
Gender		0.080	0.105		**<0.001**	0.209
Male	8.99 ± 6.17			4.36 ± 2.77		
Female	9.63 ± 6.16			4.94 ± 2.72		
Education		0.110	0.127		0.451	0.060
School level	10.06 ± 6.38			4.59 ± 2.83		
University level	9.28 ± 6.13			4.76 ± 2.74		
Smoking		**0.012**	0.151		**0.041**	0.122
No	9.06 ± 6.24			4.61 ± 2.73		
Yes	9.99 ± 6.01			4.94 ± 2.79		
Alcohol		0.123	0.149		0.340	0.092
No	9.31 ± 6.22			4.70 ± 2.73		
Yes	10.23 ± 5.69			4.96 ± 2.90		

*Note*: Numbers in bold indicate significant *p*-values.

**Table 3.** Pearson correlation matrix Table 3. long description.

	1	2	3	4	5	6
1. Depression	1					
2. Stress	0.35***	1				
3. Alexisomia	0.35***	0.21***	1			
4. Childhood abuse	0.38***	0.08**	0.06*	1		
5. Age	0.02	−0.02	0.03	0.03	1	
6. Household overcrowding index	0.05	0.06	0.04	0.01	−0.04	1

*p < .05; **p < .01; ***p < .001.

### Analysis of mediation

**Figure 1.**
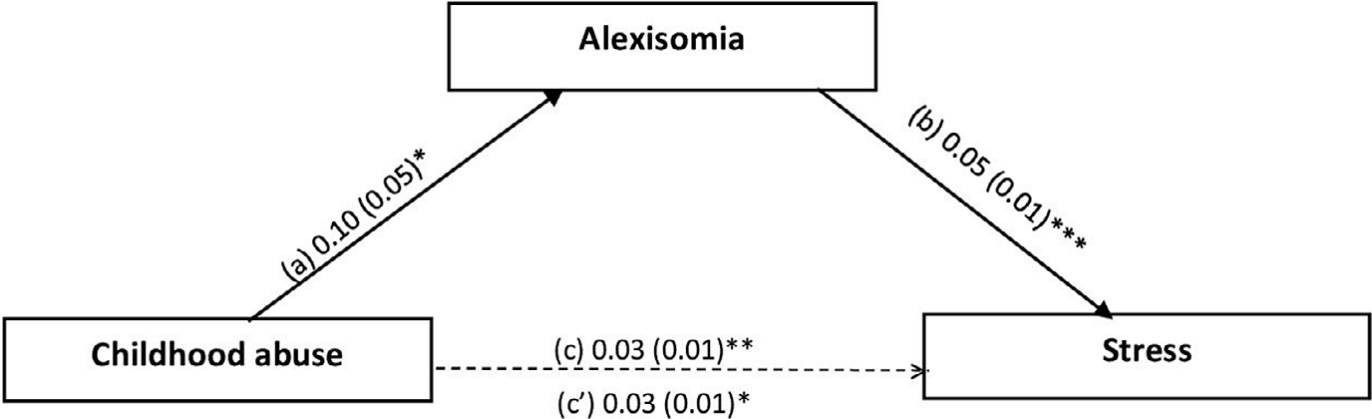
(a) Relation between childhood abuse and alexisomia (R^2^= 0.007); (b) Relation between alexisomia and stress (R^2^= 0.066); (c) Total effect of childhood abuse on stress (R^2^= 0.026); (c’) Direct effect of childhood abuse on stress. The numbers represent unstandardized regression coefficients and their standard errors. *p < 0.05; **p < .01; ***p < 0.001 Figure 1. long description.

The mediation analysis, taking stress as the dependent variable, was adjusted for the following covariates: smoking, gender and household overcrowding index. Alexisomia (indirect effect: Beta = 0.005; Boot SE = 0.002; Boot CI: 0.001–0.009) mediated the association between childhood abuse and stress (Figure 1). Higher childhood abuse was significantly associated with higher alexisomia and directly associated with higher stress. Higher alexisomia was significantly associated with higher stress (Figure 1).

**Figure 2.**
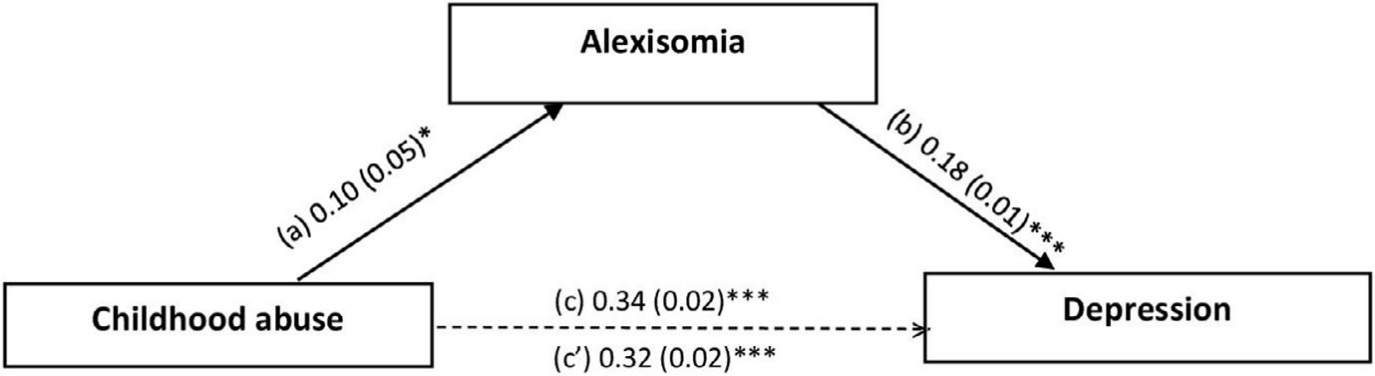
(a) Relation between childhood abuse and alexisomia (R^2^= 0.008); (b) Relation between alexisomia and depression (R^2^= 0.263); (c) Total effect of childhood abuse on depression (R^2^= 0.157); (c’) Direct effect of childhood abuse on depression. The numbers represent unstandardized regression coefficients and their standard errors. *p < 0.05; **p < .01; ***p < 0.001

The mediation analysis taking the depression score as the dependent variable was adjusted for the following covariates: smoking, gender, household overcrowding index, education and alcohol. Alexisomia (indirect effect: Beta = 0.018; Boot SE = 0.008; Boot CI: 0.002–0.034) mediated the association between childhood abuse and depression (Figure 2). Higher childhood abuse was significantly associated with higher alexisomia and directly associated with higher depression. Higher alexisomia was significantly associated with higher depression.

## Discussion

The objective of the study was to investigate the mediating role of alexisomia in the association between childhood abuse and depression/stress. The findings revealed that a history of childhood abuse was significantly associated with greater alexisomia, which, in turn, contributed to increased levels of depression and stress. Mediation analysis confirmed that alexisomia partially mediated the relationships between childhood abuse and both depression and stress, even after adjusting for covariates such as smoking, gender, household overcrowding index and education.

### Childhood abuse and depression/stress

As for the direct effect, our findings build upon previous research by demonstrating significant associations between childhood abuse and elevated levels of stress and depression, aligning with prior research (Mandelli et al., 2015; Nelson et al., 2017; Berber Çelik and Odacı, 2020; Humphreys et al., 2020; Lang et al., 2020; Li et al., 2020; Watters et al., 2021; Thurston et al., 2023). A 2023 meta-analysis found a strong association between childhood abuse and various mental health issues, such as depression, anxiety and substance use disorders (Madigan et al., 2023). This link can be explained by several mechanisms.

On a psychological level, childhood abuse fosters maladaptive cognitive schemas (Lumley and Harkness, 2007; Bing-Canar et al., 2024) – defined as broad dysfunctional patterns of cognition, emotions, memories and physical sensations related to oneself and others, and hypothesized to consolidate over time (Delcea, 2020). These schemas, shaped by repeated experiences of worthlessness, shame and fear, perpetuate depressive thinking styles (Li et al., 2020). For instance, victims of emotional abuse may internalize derogatory comments from abusers (e.g., “You are a failure”), which form the basis for negative self-associations and may lead to depressive symptoms.

On a biological level, childhood abuse has been found to dysregulate the hypothalamic–pituitary–adrenal (HPA) axis, which regulates the body’s stress-response system (Keller et al., 2017; Li et al., 2020; Madigan et al., 2023). Studies found that chronically elevated cortisol levels predispose individuals to heightened stress sensitivity, increasing the susceptibility to stress-related disorders, including PTSD (Cross et al., 2017; Keller et al., 2017).

On a social/interpersonal level, Hammen’s stress-generation model provides a useful framework for understanding this cycle (Hammen, 1991): individuals who experienced emotional abuse may engage in behaviors such as excessive reassurance-seeking to mitigate their feelings of insecurity. However, these behaviors can strain relationships, leading to rejection and further stress, which, in turn, exacerbate depressive symptoms (Braithwaite et al., 2017; Christ et al., 2019).

### Childhood abuse and alexisomia

The study revealed a significant association between childhood abuse and alexisomia. While the connection between childhood abuse and alexithymia is well established in previous research, the link to alexisomia is less frequently studied, although it is suggested (Luoni et al., 2018; Morie et al., 2020; Khan and Jaffee, 2022). On the one hand, abuse may lead to detachment from internal states as a coping mechanism to shield against overwhelming emotional pain (Doyle, 2001). Over time, this emotional disconnection could manifest as alexisomia, where individuals struggle to identify and articulate their bodily sensations. This interpretation is consistent with previous literature on interoception, defined as the sensing and processing of internal body signals (Critchley and Garfinkel, 2017). Exposure to childhood abuse may disrupt the development of interoception, leading individuals to become less aware of bodily sensations. Therefore, detachment may be a core mechanism for developing alexisomia (Hamel et al., 2024).

On the other hand, chronic parental emotional abuse can heighten children’s internal focus while simultaneously disrupting their emotional regulation skills (Cross et al., 2017). This lack of emotional regulation leaves victims unable to effectively interpret and manage their physiological states. Systematic reviews suggest that children exposed to abuse often exhibit lower extroversion and conscientiousness, impairing their ability to recognize, process and communicate physiological sensations, which further predisposes them to alexisomia (Li et al., 2020).

### Alexisomia and depression/stress

Additionally, alexisomia also resulted in higher levels of depression and stress. This finding aligns with growing evidence that difficulties in emotional awareness and somatic processing can intensify psychological distress (Honkalampi et al., 2007; Luoni et al., 2018; Khan and Jaffee, 2022; Oka and Lkhagvasuren, 2022; Xu et al., 2024). Studies reported that individuals with alexithymia are more likely to suppress emotions, leading to unresolved emotional tension and heightened stress (Morie et al., 2020).

Individuals with alexisomia struggle to process emotional and physical cues (Oka, 2020). The inability to recognize stress signals often leads to maladaptive coping strategies, such as emotional suppression and rumination, that intensify psychological distress (Flynn et al., 2010). A recent systematic review and meta-analysis found that alexithymia is consistently associated with lower interoception, including lower self-regulation and lower trust in bodily sensations (Van Bael et al., 2024). Once alexisomia develops, it perpetuates a vicious cycle: victims struggle to identify stress signals, leading to ineffective coping and heightened emotional dysregulation, potentially leading to depression and chronic stress responses (Morie et al., 2020). Moreover, alexisomia may amplify physiological stress markers, such as elevated inflammatory cytokines (Conti et al., 2017), which have been linked to both stress-related disorders and major depressive disorder (Min et al., 2023).

### Alexisomia as a mediator

Our findings demonstrate that alexisomia mediates the relationship between childhood abuse and both stress and depression. This suggests that the inability to interpret or regulate bodily feelings acts as a pathway through which early adversity contributes to psychological distress. Specifically, childhood abuse directly influences alexisomia, which, in turn, exacerbates stress and depressive symptoms. Notably, comparing our findings with prior research would be challenging, as no studies have examined the mediating role of alexisomia in the relationship between childhood abuse and depression/stress. Nevertheless, our findings are consistent with previous literature demonstrating the mediating role of alexithymia. A recent meta-analysis found alexithymia to mediate the relationship between childhood abuse and adult psychopathology (Kick et al., 2024). Furthermore, Hérbet et al. reported that alexithymia partially mediated the association between child sexual abuse and subsequent psychological distress among adolescents (Hébert et al., 2018). Taken together, these findings suggest that childhood abuse may disrupt the development of internal state awareness, which then hinders effective processing of emotional signals, thereby increasing vulnerability to stress and depressive symptoms. However, this mediation does not fully explain the relationship, suggesting additional pathways warrant exploration in future research.

### Clinical implications

Our study highlights the complex associations between childhood abuse, alexisomia, depression and stress, as well as the mediating role of alexisomia in these relationships. While the link between alexithymia and psychological distress has been extensively studied, research on alexisomia remains scarce, especially in populations with a history of childhood trauma. By revealing its connection to psychological distress, this study contributes to a deeper understanding of alexisomia and its connection with mental health outcomes such as depression and stress. Given that alexisomia is relatively underexplored, particularly in trauma-exposed individuals, our findings underscore the importance of greater clinical awareness when assessing the mental health of those with a history of abuse.

The identification of alexisomia as a key mediator in these associations highlights the potential clinical relevance of screening for emotional and physical awareness deficits in individuals at risk for trauma-related disorders, as those with alexisomia are less likely to seek help due to their impaired self-awareness.

At the clinical level, these findings can inform mental health professionals by highlighting the relevance of emotional and physical awareness deficits in individuals who have experienced childhood abuse. Mindfulness-based interventions, which have shown promise in treating alexisomia and its psychological sequelae (Amemiya and Sakairi, 2019; Liu et al., 2022), may represent a potentially useful therapeutic approach for targeting emotional and somatic awareness deficits and their associated distress. In fact, mindfulness and body-oriented interventions (e.g., yoga) have shown effectiveness in trauma-related conditions. One possible explanation is that alexisomia may constitute one of the mechanisms through which childhood trauma contributes to psychological distress. Thus, by targeting attention to bodily sensations and interoception – central concepts of alexisomia – these techniques may help modulate psychological distress in patients with childhood abuse. Given the significant impact of alexisomia on mental health outcomes in this study, future research should further explore targeted interventions aimed at both alexisomia and childhood trauma. Longitudinal studies would be particularly valuable in understanding the long-term effects of these interventions and their potential to prevent the development of more severe psychological conditions. Moreover, additional research is needed to fully unravel the mediation process between childhood abuse and mental health outcomes, as the current understanding remains incomplete. Alternative mediation models should also be explored in future research. The present study found an association between childhood abuse and alexisomia. Given that childhood abuse is associated with depression and stress (Hoying et al., 2020), and that these conditions have also been linked to impairment in somatic awareness (Güleç et al., 2013; Oka, 2020), depression and stress may mediate the relationship between childhood abuse and alexisomia by increasing difficulties in interpreting bodily sensations. This could ultimately inform more effective, evidence-based treatments for individuals affected by childhood trauma.

### Limitations

Several limitations of this study should be considered when interpreting the findings. The cross-sectional design precludes the establishment of causal relationships, limiting the ability to infer directional or temporal associations. Additionally, the reliance on self-reported data introduces potential biases, including recall bias and social desirability bias, which may affect the accuracy of participants’ responses.

There is also a potential for residual confounding bias, as not all factors associated with depression and stress were included in the analysis (e.g., lifestyle including physical activity and diet, genetic predispositions or comorbid physical health conditions). Furthermore, the snowball sampling technique used for data collection reduces the randomness of the sample and constitutes a sampling bias. This limitation affects the ability to generalize the results to broader populations, such as individuals with histories of childhood trauma or other specific subgroups.

Despite these constraints, the study provides valuable insights into the associations between childhood abuse, alexisomia and mental health outcomes, paving the way for further research in this area.

## Conclusion

The findings of this study provide evidence for the association between childhood abuse and both depression and stress, highlighting the role of alexisomia as a partial mediator in these relationships. Enhancing emotional and physical awareness through targeted interventions may help reduce the impact of childhood abuse on mental health, ultimately reducing stress and depression levels and improving the overall well-being of affected individuals. Greater clinical awareness of alexisomia, particularly in populations with a history of trauma, can aid in refining diagnostic and therapeutic approaches to address these challenges. Future research exploring tailored interventions that specifically target alexisomia may offer promising strategies for diminishing the long-term consequences of childhood trauma on mental health.

## Data Availability

The data that support the findings of this study are available from the corresponding author, but restrictions apply to the availability of these data, which were used under license for the current study, and so are not publicly available. Data are, however, available from the authors upon reasonable request and with permission of the ethics committee.

## Long descriptions

### Table 1. Long description

The table is divided into two sections.

The first section lists categorical variables with N and percentages:

* Gender: Male 437 (36.3 percent), Female 768 (63.7 percent).

* Education: School level 188 (15.6 percent), University level 1,017 (84.4 percent).

* Current smoking: No 766 (63.6 percent), Yes 439 (36.4 percent).

* Current alcohol drinking: No 1,086 (90.1 percent), Yes 119 (9.9 percent).

The second section lists continuous variables with Mean plus or minus S D:

* Age (years): 27.16 plus or minus 10.23.

* Household overcrowding index (person/room): 1.12 plus or minus 0.59.

* Childhood abuse: 10.17 plus or minus 7.01.

* Depression: 9.40 plus or minus 6.17.

* Stress: 4.73 plus or minus 2.75.

* Alexisomia: 49.26 plus or minus 11.39.

Navigate back to Table 1..

### Table 2. Long description

A statistical table with seven columns. The first column lists factors, followed by two main sections: Depression score and Stress score. Each section includes three columns: Mean plus or minus S D, p, and Effect size.

* Gender: For Depression, p equals 0.080 and Effect size is 0.105. Male mean is 8.99 plus or minus 6.17; Female mean is 9.63 plus or minus 6.16. For Stress, p is less than 0.001 (significant) and Effect size is 0.209. Male mean is 4.36 plus or minus 2.77; Female mean is 4.94 plus or minus 2.72.

* Education: For Depression, p equals 0.110 and Effect size is 0.127. School level mean is 10.06 plus or minus 6.38; University level mean is 9.28 plus or minus 6.13. For Stress, p equals 0.451 and Effect size is 0.060. School level mean is 4.59 plus or minus 2.83; University level mean is 4.76 plus or minus 2.74.

* Smoking: For Depression, p equals 0.012 (significant) and Effect size is 0.151. No mean is 9.06 plus or minus 6.24; Yes mean is 9.99 plus or minus 6.01. For Stress, p equals 0.041 (significant) and Effect size is 0.122. No mean is 4.61 plus or minus 2.73; Yes mean is 4.94 plus or minus 2.79.

* Alcohol: For Depression, p equals 0.123 and Effect size is 0.149. No mean is 9.31 plus or minus 6.22; Yes mean is 10.23 plus or minus 5.69. For Stress, p equals 0.340 and Effect size is 0.092. No mean is 4.70 plus or minus 2.73; Yes mean is 4.96 plus or minus 2.90.

Navigate back to Table 2..

### Table 3. Long description

The table presents correlation coefficients for six variables. The diagonal values are all 1.

* Depression (Variable 1) correlates with Stress at 0.35, Alexisomia at 0.35, and Childhood abuse at 0.38. All three are significant at p < .001. Its correlations with Age (0.02) and Household overcrowding index (0.05) are not significant.

* Stress (Variable 2) correlates with Alexisomia at 0.21 (p < .001) and Childhood abuse at 0.08 (p < .01). Its correlations with Age (-0.02) and Household overcrowding index (0.06) are not significant.

* Alexisomia (Variable 3) correlates with Childhood abuse at 0.06 (p < .05). Its correlations with Age (0.03) and Household overcrowding index (0.04) are not significant.

* Childhood abuse (Variable 4) has non-significant correlations with Age (0.03) and Household overcrowding index (0.01).

* Age (Variable 5) has a non-significant correlation with Household overcrowding index at -0.04.

Significance levels are indicated by asterisks: * p < .05; ** p < .01; *** p < .001.

Navigate back to Table 3..

### Figure 1. Long description

The flowchart consists of three rectangular boxes connected by arrows.

* At the bottom-left is a box labeled Childhood abuse.

* At the top-center is a box labeled Alexisomia.

* At the bottom-right is a box labeled Stress.

Pathways and statistical values:

* A solid arrow points from Childhood abuse up to Alexisomia, labeled (a) 0.10 (0.05)*.

* A solid arrow points from Alexisomia down to Stress, labeled (b) 0.05 (0.01)***.

* A dashed horizontal arrow points directly from Childhood abuse to Stress. It is associated with two sets of values: (c) 0.03 (0.01)** and (c’) 0.03 (0.01)*.

Navigate back to Figure 1..
