# Supplementary material for: The mediating effect of alexisomia between childhood abuse and depression/stress in a sample of Lebanese adults: A cross-sectional study
Source: Glob Ment Health (Camb). 2026 Jul 29;13:e165. doi: 10.1017/gmh.2026.10275 (PMC13433335; doi:10.1017/gmh.2026.10275)
Supplement: Peer review history

# Reviewer report: Review: The mediating effect of alexisomia between childhood abuse and depression/stress in a sample of Lebanese adults: A cross-sectional study — R0/PR2

**DOI:** 10.1017/gmh.2026.10275.pr2

**Authors:** Efruz Pirdogan Aydin

This manuscript examines the mediating role of alexisomia in the relationship between childhood abuse and depression/stress in a Lebanese adult sample. The topic is relevant and potentially contributes to the literature by focusing on a relatively understudied construct related to bodily awareness. Exploring alexisomia as a possible mechanism linking early adverse experiences to psychological distress may provide clinically meaningful insights. The relatively large sample size is also a strength of the study. However, several conceptual and interpretative issues should be addressed in order to improve the clarity and robustness of the manuscript.

Introduction

• Although alexisomia is the central construct of the study, it is introduced relatively late in the Introduction. The rationale for selecting alexisomia specifically as a mediator should be more clearly and more strongly justified.

• The section describing general background information on depression and stress appears somewhat lengthy. This part could be shortened so that the research gap and the novelty of the study become evident earlier.

• Terminology should be used consistently throughout the manuscript. The expressions “child abuse” and “childhood abuse” are used interchangeably and would benefit from standardization.

Methods and Results

• Although the findings are statistically significant, the association between child abuse and alexisomia appears relatively weak and this should be more clearly acknowledged.

• The proportion of explained variance for alexisomia is quite low and this should be taken into consideration when interpreting the findings.

• The indirect effects are statistically significant, but the effect appear small; therefore, the Results section would benefit from a more balanced interpretation of these effects.

• It should be clarified whether the coefficients presented in the tables and figures are standardized or unstandardized.

Discussion

• The Discussion should avoid wording that implies causality, as the cross-sectional design allows only statistical mediation, not causal inference.

• The clinical significance of alexisomia should be discussed more cautiously, particularly given the relatively small effect sizes.

• The findings could be more strongly integrated with the literature on alexithymia, interoception, and somatic awareness.

• Based on the correlation pattern, alternative mediation models may also be theoretically plausible. In particular, models in which depression or stress are specified as mediators and alexisomia as the outcome could be informative. Testing such alternative models could strengthen the manuscript. If these analyses are not conducted, these alternative directional explanations should at least be acknowledged more explicitly in the Discussion.

# Reviewer report: Review: The mediating effect of alexisomia between childhood abuse and depression/stress in a sample of Lebanese adults: A cross-sectional study — R0/PR3

**DOI:** 10.1017/gmh.2026.10275.pr3

**Authors:** Jana Javakhishvili

**Affiliations:** School of Arts and Science, Ilia State University, Georgia

Review of the paper “The Mediating Effect of Alexisomia Between Child Abuse and Depression/Stress in a Sample of Lebanese Adults: A Cross-Sectional Study”

This study examines the relationship between childhood abuse and depression and stress in adulthood, with a particular focus on the mediating role of alexisomia. The topic is relevant, especially given the focus on a war-affected and economically and socio-politically disadvantaged country, and its implications for clinical practice. A notable strength of the paper is its effort to explore the understudied within the context of the traumatic stress phenomenon of alexisomia.

Introduction

- The introduction would benefit from being substantially shortened and more focused. At present, it places considerable emphasis on well-established relationships (e.g., between adverse childhood experiences, depression, and stress), while the relatively novel construct of alexisomia receives equal (if not less) attention. Rebalancing the section to foreground alexisomia would better highlight the study’s contribution.

- A more explicit integration of trauma-awareness and Adverse Childhood Experiences (ACE) awareness perspectives would also strengthen the theoretical framing. For example, stress should not only be presented as an outcome but also as a potential causal pathway to depression, particularly in the context of exposure to potentially traumatic stressors such as childhood abuse. Similarly, the discussion of violence and trauma could more clearly acknowledge traumatic stress and its well-documented comorbidity with depression and PTSD.

- There are also several points requiring clarification or refinement:

* The order of conditions listed (p. 4, lines 29–32) would be more appropriate if “chronic illnesses” preceded “suicide.”

* Greater consistency in terminology is needed throughout (e.g., earlier introduction and consistent use of “trauma,” “distress,” and Adverse Childhood Experiences “ACE"; now they are just occasionally popping up in the text, Complex Post-Traumatic Stress Disorder (CPTSD) is completely missing).

* Throughout the paper, the terms “child abuse” and “childhood abuse” are used interchangeably, although they are not synonymous. This should be corrected throughout for precision. More generally, the manuscript would benefit from careful language editing to improve clarity and consistency.

* The relationship between alexisomia and alexithymia should be clarified when both terms are introduced.

Overall, the introduction currently reads more like an extended thesis-style overview; a more concise, targeted, and conceptually integrated version would strengthen the paper.

Methods

Some information is unnecessarily repeated (e.g., description of the questionnaire as online, anonymous, and self-administered). Reducing such repetitions throughout the paper would improve readability. It would also be helpful to:

- Introduce the original authors of each measure when first mentioned, and follow a consistent referencing style throughout.

- Apply this consistently across all instruments (e.g., CASRS-12, STSS, PHQ-9, SNRS-11).

- Provide a clearer description of the SNRS-11 scale: what dimensions of stress it measures, and whether it captures subjective distress or exposure to stressors. The authorship of this measure should also be specified.

In the subsection concerning ethics, it should be clearly stated whether the study received approval from an ethics review board.

Results

- When reporting the mean age of participants, the standard deviation should also be included.

- The inclusion of alcohol and tobacco use within demographic characteristics requires clarification. It is unclear what is meant by these variables (e.g., lifetime use, past-year use, daily use). Without such specification, the findings are difficult to interpret.

- Minor stylistic improvements are also needed (e.g., avoiding consecutive use of “furthermore” and “moreover”).

Discussion

- The discussion would benefit from consistent use of the term “childhood abuse.”

- The section on therapeutic implications is valuable; however, the discussion of mindfulness could be further enriched by considering how alexisomia may help explain the effectiveness of mindfulness-based and body-oriented interventions (e.g., yoga) in trauma-related conditions.

Limitations

Sampling bias related to the use of snowball sampling should be explicitly acknowledged.

Conclusions

There is an inconsistency between the Discussion, Results, and Conclusions sections: the Discussion refers to partial mediation, whereas this qualifier is omitted in the Conclusions and is not mentioned in the corresponding narrative paragraphs of the Results section. For accuracy and consistency, this should be retained, as it reflects the fact that childhood abuse may also contribute to depression and distress independently of alexisomia.

Overall evaluation

This is an interesting and promising study addressing an important topic. With revisions to improve focus, conceptual clarity, terminology, and consistency, the manuscript has strong potential. I would like to congratulate the authors on their important work and thank the editor for the opportunity to review this interesting paper.

# Editor report: Recommendation: The mediating effect of alexisomia between childhood abuse and depression/stress in a sample of Lebanese adults: A cross-sectional study — R0/PR4

**DOI:** 10.1017/gmh.2026.10275.pr4

**Affiliations:** Faculty of Business, Technology, and Education, Ilia State University, Georgia

Dear Authors, 

We have now received the reviewers’ comments on your manuscript.

Please carefully review all feedback and prepare a detailed, point-by-point response addressing each comment raised by the reviewers. For clarity and efficiency in the next evaluation round, we ask that you:

• Provide a structured response to every comment (indicating where changes were made or explaining when no change was made).

• Highlight all revisions in the manuscript (e.g., using track changes or colored text).

• Ensure that your responses are precise, evidence-based, and professionally framed.

Kindly submit your revised manuscript along with the response-to-reviewers document. If any comments require clarification or if you anticipate delays, please inform us promptly.

We look forward to receiving your revision.

Best regards,

N.Makhashvili

# Editor report: Decision: The mediating effect of alexisomia between childhood abuse and depression/stress in a sample of Lebanese adults: A cross-sectional study — R0/PR5

**DOI:** 10.1017/gmh.2026.10275.pr5

**Affiliations:** Johns Hopkins University Bloomberg School of Public Health, United States

No accompanying comment.

# Reviewer report: Review: The mediating effect of alexisomia between childhood abuse and depression/stress in a sample of Lebanese adults: A cross-sectional study — R1/PR7

**DOI:** 10.1017/gmh.2026.10275.pr7

**Authors:** Efruz Pirdogan Aydin

The authors have satisfactorily addressed this concern, and the manuscript is stronger in its revised form.

# Editor report: Recommendation: The mediating effect of alexisomia between childhood abuse and depression/stress in a sample of Lebanese adults: A cross-sectional study — R1/PR8

**DOI:** 10.1017/gmh.2026.10275.pr8

**Affiliations:** Faculty of Business, Technology, and Education, Ilia State University, Georgia

No accompanying comment.

# Editor report: Decision: The mediating effect of alexisomia between childhood abuse and depression/stress in a sample of Lebanese adults: A cross-sectional study — R1/PR9

**DOI:** 10.1017/gmh.2026.10275.pr9

**Affiliations:** Johns Hopkins University Bloomberg School of Public Health, United States

No accompanying comment.
